# Supplementary material for: Screening of Helicoverpa armigera Mobilome Revealed Transposable Element Insertions in Insecticide Resistance Genes
Source: Insects. 2020 Dec 11;11(12):879. doi: 10.3390/insects11120879 (PMC7764229; doi:10.3390/insects11120879)
Supplement: Supplementary file 1 [file insects-11-00879-s001.zip › Figure S1-S7.docx]

LINE_R2_526 ------------------------------------------------------------ 0

LOC110375617 AAGCCTGTATGTATAAAAACTTATTTTATGCTTCGTAATGGACTAGTTGGTCAGTGGAAT 60

LINE_R2_526 ------------------------------------------------------------ 0

LOC110375617 GGTAAATACCATGTCCATAGCCGTGCGTGGAATTCATTTCATATAGTTCACAAAGAGGTT 120

LINE_R2_526 ------------------------------------------------------------ 0

LOC110375617 GTATAGTGACCATACTTGCTTTAAGAGTAGCGCAGTGAAAAGGCCTAATACGGAAAGCCT 180

[….]

LINE_R2_526 ------------------------------------------------------------ 0

LOC110375617 TGGGGGGGCTTAGAGGAGGCATGAGCAATTAATATAGAGGTCCTAACAACTAGACCCACC 6480

LINE_R2_526 --TGGAATATAGCCAGGAGGTACGAAATCAGTCACTAACACCTTGACACACCCTTCCAAC 58

LOC110375617 CCTGGAATATAGCCAGGAGGTACGAAATCAGTCACTAACACCTTGACACACCCTTCCAAC 6540

**********************************************************

LINE_R2_526 TAACGGTCCCAAACCAACCCCTCCAATATGGAAACCAGATCTAGAAAGAAACGTATAGGG 118

LOC110375617 TAACGGTCCCAAACCAACCCCTCCAATATGGAAACCAGATCTAGAAAGAAACGTATAGGG 6600

************************************************************

LINE_R2_526 CCGCTGCCCGGGGGCGATCGCCGGGGCACACCTGGAGCTGACGCTGGGTGTCTCAGCATG 178

LOC110375617 CCGCTGCCCGGGGGCGATCGCCGGGGCACACCTGGAGCTGACGCTGGGTGTCTCAGCATG 6660

************************************************************

LINE_R2_526 CGATCCGTCAGTGGTGAGGAGTTGAGCAGGCGGGCTCCCACCATGCAATATGCTACAGCC 238

LOC110375617 CGATCCGTCAGTGGTGAGGAGTTGAGCAGGCGGGCTCCCACCATGCAATATGCTACAGCC 6720

************************************************************

LINE_R2_526 GACGAAGAAGTAGATAGGATAAATAGCCCATTAGAACCATTCAGTCCAAGCACCGCTTCT 298

LOC110375617 GACGAAGAAGTAGATAGGATAAATAGCCCATTAGAACCATTCAGTCCAAGCACCGCTTCT 6780

************************************************************

LINE_R2_526 TATGTCCCGTCACTATCATCATCCCCCTCCACAGTTAGTTTAAATGCCGAAAGTATAGTT 358

LOC110375617 TATGTCCCGTCACTATCATCATCCCCCTCCACAGTTAGTTTAAATGCCGAAAGTATAGTT 6840

************************************************************

LINE_R2_526 TTTCACCAACATGTATCATCTGATACGCCCACGCCTGAGGATGTTGTGCAGGAGGCACTC 418

LOC110375617 TTTCACCAACATGTATCATCTGATACGCCCACGCCTGAGGATGTTGTGCAGGAGGCACTC 6900

************************************************************

LINE_R2_526 GCCACTGAGCTTGCACCCGCCCCAGGCACAAGTCGTGCGCGCAAACGATGGTCAGAAGAA 478

LOC110375617 GCCACTGAGCTTGCACCCGCCCCAGGCACAAGTCGTGCGCGCAAACGATGGTCAGAAGAA 6960

************************************************************

LINE_R2_526 ATGAATAAATTCATCTGGCGCACGTACCTAATAGCTACAAAACTGAATACAAATAAAATT 538

LOC110375617 ATGAATAAATTCATCTGGCGCACGTACCTAATAGCTACAAAACTGAATACAAATAAAATT 7020

************************************************************

LINE_R2_526 TATCTCCAACAACTCCATACCGAATTCTCGTTAAAATTCCCTGATATGCAAGCATCTAGA 598

LOC110375617 TATCTCCAACAACTCCATACCGAATTCTCGTTAAAATTCCCTGATATGCAAGCATCTAGA 7080

************************************************************

LINE_R2_526 CAAAGACTCGGAGATCAATGTAGGGCCATAGTAAGAAATAAATTACTGCCACAGGAAACA 658

LOC110375617 CAAAGACTCGGAGATCAATGTAGGGCCATAGTAAGAAATAAATTACTGCCACAGGAAACA 7140

************************************************************

LINE_R2_526 CTTGACCAAATAAGGGAAGAAGTCACAACTCATCTACAAACAAACAGCACACTACAAACT 718

LOC110375617 CTTGACCAAATAAGGGAAGAAGTCACAACTCATCTACAAACAAACAGCACACTACAAACT 7200

************************************************************

LINE_R2_526 CACACAGAAACAAATACACAAATACACACAAATTCTCAGCAAGTGGGTGGACAGAGACGA 778

LOC110375617 CACACAGAAACAAATACACAAATACACACAAATTCTCAGCAAGTGGGTGGACAGAGACGA 7260

************************************************************

LINE_R2_526 AGATGGACTACTGAACAAAATGAATCAATTATTAGACACTACTATAAAATAACAGCGATG 838

LOC110375617 AGATGGACTACTGAACAAAATGAATCAATTATTAGACACTACTATAAAATAACAGCGATG 7320

************************************************************

LINE_R2_526 GAACAAAATAGATCAGCTTATCGTCAACCTCTCCATCAGGCAGTCATAACAGAACATCCA 898

LOC110375617 GAACAAAATAGATCAGCTTATCGTCAACCTCTCCATCAGGCAGTCATAACAGAACATCCA 7380

************************************************************

LINE_R2_526 GAACTAAGTGGTGTAACAGAACAGAGAATCTCAGACCAACTACGGGTAATATTGAATAAC 958

LOC110375617 GAACTAAGTGGTGTAACAGAACAGAGAATCTCAGACCAACTACGGGTAATATTGAATAAC 7440

************************************************************

LINE_R2_526 AAAATGATAACCGACCAGAGGCTCGACGAAATACGAAGTGAGATTGCCCAGGAAATTAAC 1018

LOC110375617 AAAATGATAACCGACCAGAGGCTCGACGAAATACGAAGTGAGATTGCCCAGGAAATTAAC 7500

************************************************************

LINE_R2_526 TCTTACAATAACGAAAATATCACGCTGCCAACAACGCAAAACGGAACACACACATCTCAG 1078

LOC110375617 TCTTACAATAACGAAAATATCACGCTGCCAACAACGCAAAACGGAACACACACATCTCAG 7560

************************************************************

LINE_R2_526 TCAGAAGACACCCAAGAAACACAAATGCACTTGTTACAAAATATACCTTCACATGAATTG 1138

LOC110375617 TCAGAAGACACCCAAGAAACACAAATGCACTTGTTACAAAATATACCTTCACATGAATTG 7620

************************************************************

LINE_R2_526 AATCTATCAATACACACGCAGTCACATTCCCTCCCAACAGATAGTCAGAACCCAGAACTT 1198

LOC110375617 AATCTATCAATACACACGCAGTCACATTCCCTCCCAACAGATAGTCAGAACCCAGAACTT 7680

************************************************************

LINE_R2_526 CATCAAAATCAATTACCTGATACCGATGATTTAGAGAAAATTAAAGATCAATTCAAAAAC 1258

LOC110375617 CATCAAAATCAATTACCTGATACCGATGATTTAGAGAAAATTAAAGATCAATTCAAAAAC 7740

************************************************************

LINE_R2_526 ACTTTTGATAGGTTTCGTAACACTAGTCCTACCGACCGACCTTATATTCCAAAGCAAAAA 1318

LOC110375617 ACTTTTGATAGGTTTCGTAACACTAGTCCTACCGACCGACCTTATATTCCAAAGCAAAAA 7800

************************************************************

LINE_R2_526 ACCTCTCGAAGACTAGCTGTTATTGTAGATGTAATAAACACCAAAATACTTCCTCAATAC 1378

LOC110375617 ACCTCTCGAAGACTAGCTGTTATTGTAGATGTAATAAACACCAAAATACTTCCTCAATAC 7860

************************************************************

LINE_R2_526 ATTGCAAGAGATCAAGATTTCGAAACAACACATACAATCACCTATTGTGCAGCCTACACA 1438

LOC110375617 ATTGCAAGAGATCAAGATTTCGAAACAACACATACAATCACCTATTGTGCAGCCTACACA 7920

************************************************************

LINE_R2_526 GCTGCTATCTGTAACGGAGCTAAAATCAGAGAATCTGTTCTACCTACCCAGCCCACACAA 1498

LOC110375617 GCTGCTATCTGTAACGGAGCTAAAATCAGAGAATCTGTTCTACCTACCCAGCCCACACAA 7980

************************************************************

LINE_R2_526 ACAAGAAAACCCGCGTGGCAGAAGAGACTAGAGAAAAAGATAGAGTCTCTGAGAAGAGAT 1558

LOC110375617 ACAAGAAAACCCGCGTGGCAGAAGAGACTAGAGAAAAAGATAGAGTCTCTGAGAAGAGAT 8040

************************************************************

LINE_R2_526 ATTGCTAGGCTGTCTGAATATGCAAGGGGAACTAGAAGCAATAGACTAATCTTACTAATA 1618

LOC110375617 ATTGCTAGGCTGTCTGAATATGCAAGGGGAACTAGAAGCAATAGACTAATCTTACTAATA 8100

************************************************************

LINE_R2_526 GAGATAATAAAAAACAAATATCGTATACACTCCCAACATGAAGAACCCAACTTAAGCAAT 1678

LOC110375617 GAGATAATAAAAAACAAATATCGTATACACTCCCAACATGAAGAACCCAACTTAAGCAAT 8160

************************************************************

LINE_R2_526 GATGATTATCTCGACACACTCAAACAAAAACTTAATGCAGCTTGTAGTAGATTAAAAAGA 1738

LOC110375617 GATGATTATCTCGACACACTCAAACAAAAACTTAATGCAGCTTGTAGTAGATTAAAAAGA 8220

************************************************************

LINE_R2_526 TATGTTACCTGTACTTTACGAAAGAAACAAAACTCCCGATTTGTTAATAATGAAAAGCAA 1798

LOC110375617 TATGTTACCTGTACTTTACGAAAGAAACAAAACTCCCGATTTGTTAATAATGAAAAGCAA 8280

************************************************************

LINE_R2_526 TTCTATAGAACACTCGCATCAAACACACAGAATAACACAAGCACAGAGGCACAAGAGATA 1858

LOC110375617 TTCTATAGAACACTCGCATCAAACACACAGAATAACACAAGCACAGAGGCACAAGAGATA 8340

************************************************************

LINE_R2_526 CTAACACCACCCGCAGAGGAACTTCATAGGTTTTGGTCAGGGATCTGGGCAGAGCCAGTA 1918

LOC110375617 CTAACACCACCCGCAGAGGAACTTCATAGGTTTTGGTCAGGGATCTGGGCAGAGCCAGTA 8400

************************************************************

LINE_R2_526 CAACATAATGCAGACGCTGAATGGTTAGACGCAGACGCTGACATAGTTGATAAAATTACA 1978

LOC110375617 CAACATAATGCAGACGCTGAATGGTTAGACGCAGACGCTGACATAGTTGATAAAATTACA 8460

************************************************************

LINE_R2_526 CCGATGCAATTTGATCATATACCAATAGATGTATTCGTGACAGTTCTTTGTAAAGCTCAT 2038

LOC110375617 CCGATGCAATTTGATCATATACCAATAGATGTATTCGTGACAGTTCTTTGTAAAGCTCAT 8520

************************************************************

LINE_R2_526 AACTGGAAAGCACCTGGTAGTGATCGAATACACAACTATTGGTATAAGAAACTTACAATC 2098

LOC110375617 AACTGGAAAGCACCTGGTAGTGATCGAATACACAACTATTGGTATAAGAAACTTACAATC 8580

************************************************************

LINE_R2_526 CTACACCCTTTACTCCACAACCACATAAATAATTTCGTTCAGACTCCAAATTCTATGCCC 2158

LOC110375617 CTACACCCTTTACTCCACAACCACATAAATAATTTCGTTCAGACTCCAAATTCTATGCCC 8640

************************************************************

LINE_R2_526 CACTTTGTCACGCAAGGCTTAACTTACCTGATACCAAAGGATAGCGACTACCAAAATCCA 2218

LOC110375617 CACTTTGTCACGCAAGGCTTAACTTACCTGATACCAAAGGATAGCGACTACCAAAATCCA 8700

************************************************************

LINE_R2_526 GCAAAATATCGTCCCATAACCTGCCTACAAACAATATATAAAATACTTACAGGATGCATT 2278

LOC110375617 GCAAAATATCGTCCCATAACCTGCCTACAAACAATATATAAAATACTTACAGGATGCATT 8760

************************************************************

LINE_R2_526 GCAGAACTTTTACACCAACATATCACTATAGATAATAACATTCTCGCAGAGGAGCAAAAA 2338

LOC110375617 GCAGAACTTTTACACCAACATATCACTATAGATAATAACATTCTCGCAGAGGAGCAAAAA 8820

************************************************************

LINE_R2_526 GGCTGCCGCAAGAGTAGCCAGGGTTGTAAAGAACAGTTAATAATAGATTCCGTTGCCATG 2398

LOC110375617 GGCTGCCGCAAGAGTAGCCAGGGTTGTAAAGAACAGTTAATAATAGATTCCGTTGCCATG 8880

************************************************************

LINE_R2_526 AAAACTGCCTTCAGCAAGAAGAAGAATATAAACACAATGTATATTGATTACAAGAAAGCT 2458

LOC110375617 AAAACTGCCTTCAGCAAGAAGAAGAATATAAACACAATGTATATTGATTACAAGAAAGCT 8940

************************************************************

LINE_R2_526 TTTGATTCCGTTCCGCATAGCTGGCTTTTAAACATCCTTCATCGCTATAAAATACACCCA 2518

LOC110375617 TTTGATTCCGTTCCGCATAGCTGGCTTTTAAACATCCTTCATCGCTATAAAATACACCCA 9000

************************************************************

LINE_R2_526 CAAATAACCAACTTCCTTGAGAACTCCATGAAACATTGGACCACGACCTTAAAAACATTA 2578

LOC110375617 CAAATAACCAACTTCCTTGAGAACTCCATGAAACATTGGACCACGACCTTAAAAACATTA 9060

************************************************************

LINE_R2_526 GGACCTAACACTACGGCTACAAACGACATTCCGATACGTAGGGGCATTTTCCAAGGGGAT 2638

LOC110375617 GGACCTAACACTACGGCTACAAACGACATTCCGATACGTAGGGGCATTTTCCAAGGGGAT 9120

************************************************************

LINE_R2_526 GCCCTGAGCCCTCTGTGGTTTTGCCTTGCACTAAATCCGCTCTCCCATATGCTGAATAAA 2698

LOC110375617 GCCCTGAGCCCTCTGTGGTTTTGCCTTGCACTAAATCCGCTCTCCCATATGCTGAATAAA 9180

************************************************************

LINE_R2_526 TCTCAAATCGGTTACACCATTAAGACACCACAAAAACACACCAATCTTACTCACCTCATG 2758

LOC110375617 TCTCAAATCGGTTACACCATTAAGACACCACAAAAACACACCAATCTTACTCACCTCATG 9240

************************************************************

LINE_R2_526 TACATGGACGACATAAAACTATACAGTGACACAACACACTCGCTACATCGCCTTGCAGAC 2818

LOC110375617 TACATGGACGACATAAAACTATACAGTGACACAACACACTCGCTACATCGCCTTGCAGAC 9300

************************************************************

LINE_R2_526 ATCACTCAATCTTTCTCCAACGATATACACATGGAATTCGGAATCGATAAATGCAAGACC 2878

LOC110375617 ATCACTCAATCTTTCTCCAACGATATACACATGGAATTCGGAATCGATAAATGCAAGACC 9360

************************************************************

LINE_R2_526 TTCTCTGTCAGCAGCGGCAAAATAACCGAAAACAGTTACACTCTAGATTCAGGCAACATA 2938

LOC110375617 TTCTCTGTCAGCAGCGGCAAAATAACCGAAAACAGTTACACTCTAGATTCAGGCAACATA 9420

************************************************************

LINE_R2_526 ATCGAACCGTTAGAACCACACACAACATACAAATATTTAGGCTTCCAACAAGCTAGACAG 2998

LOC110375617 ATCGAACCGTTAGAACCACACACAACATACAAATATTTAGGCTTCCAACAAGCTAGACAG 9480

************************************************************

LINE_R2_526 ATCAACCAAAAAGAAACAAAAGACAACCTGAAAAAGAAATTTAAGCACCGCCTAAATACC 3058

LOC110375617 ATCAACCAAAAAGAAACAAAAGACAACCTGAAAAAGAAATTTAAGCACCGCCTAAATACC 9540

************************************************************

LINE_R2_526 ATAAGCAGATCTCAATTAAATTCACGCAACTCATCTAAAGCCATTAATAGCTTCGCTATC 3118

LOC110375617 ATAAGCAGATCTCAATTAAATTCACGCAACTCATCTAAAGCCATTAATAGCTTCGCTATC 9600

************************************************************

LINE_R2_526 CCCGTCCTCACCTACTCGTTTGGGATAATCAACTGGTCCCAAGGCGACCTACTTAACCTG 3178

LOC110375617 CCCGTCCTCACCTACTCGTTTGGGATAATCAACTGGTCCCAAGGCGACCTACTTAACCTG 9660

************************************************************

LINE_R2_526 CAAAGAGTCATAAACACCACTCTCACAGCTCACCGGAAGCACCATCCAAGATCTTGTGTG 3238

LOC110375617 CAAAGAGTCATAAACACCACTCTCACAGCTCACCGGAAGCACCATCCAAGATCTTGTGTG 9720

************************************************************

LINE_R2_526 CAAAGGATGACTCTCCCAAGACGTGAAGGCGGAAGGGGAATCATAGACGTTGTTAATCTA 3298

LOC110375617 CAAAGGATGACTCTCCCAAGACGTGAAGGCGGAAGGGGAATCATAGACGTTGTTAATCTA 9780

************************************************************

LINE_R2_526 CACAATAGACAGATCACCAATCTTAGACAATATTTCTACCATCATTCAGAACACTCAACA 3358

LOC110375617 CACAATAGACAGATCACCAATCTTAGACAATATTTCTACCATCATTCAGAACACTCAACA 9840

************************************************************

LINE_R2_526 CTACACGAAACTGTAACCCTCGCTGATACCCACCTAACTCCCTTGAACCTAGCCGATCGA 3418

LOC110375617 CTACACGAAACTGTAACCCTCGCTGATACCCACCTAACTCCCTTGAACCTAGCCGATCGA 9900

************************************************************

LINE_R2_526 AACCCGCAGAAGAATGAGAAAATTACCGATGCAAAGGAGAAGATCGCCACATGGAGGCAG 3478

LOC110375617 AACCCGCAGAAGAATGAGAAAATTACCGATGCAAAGGAGAAGATCGCCACATGGAGGCAG 9960

************************************************************

LINE_R2_526 AAGTCTCTACACGGGCGGCACTACCACGACCTGCAGCAACCCCATGTCGACAAGAATGCG 3538

LOC110375617 AAGTCTCTACACGGGCGGCACTACCACGACCTGCAGCAACCCCATGTCGACAAGAATGCG 10020

************************************************************

LINE_R2_526 TCGAACGCCTGGCTGCAGCGTGGTGAGCTGTTCCCTGAGACGGAAGCCTTCATGATGGCA 3598

LOC110375617 TCGAACGCCTGGCTGCAGCGTGGTGAGCTGTTCCCTGAGACGGAAGCCTTCATGATGGCA 10080

************************************************************

LINE_R2_526 ATTCAAGACCAGGTCATCGATACCCGCAATTACCAAAAACACATAATGCGAGTCCGTAAC 3658

LOC110375617 ATTCAAGACCAGGTCATCGATACCCGCAATTACCAAAAACACATAATGCGAGTCCGTAAC 10140

************************************************************

LINE_R2_526 CTCCCCACAGATGCATGCCGACACTGCCACTCATCCCCTGAAACCATTCAACACATTACG 3718

LOC110375617 CTCCCCACAGATGCATGCCGACACTGCCACTCATCCCCTGAAACCATTCAACACATTACG 10200

************************************************************

LINE_R2_526 TCTGCCTGCAGATCTCTGGCGCAAACAGATTATAAACACCGCCATGATCAAGTAGCATCA 3778

LOC110375617 TCTGCCTGCAGATCTCTGGCGCAAACAGATTATAAACACCGCCATGATCAAGTAGCATCA 10260

************************************************************

LINE_R2_526 ATAGTCCACCAGCATCTCGCTCATAAACTTAAATTCATTGACAAAAAAATAGCCTATTAC 3838

LOC110375617 ATAGTCCACCAGCATCTCGCTCATAAACTTAAATTCATTGACAAAAAAATAGCCTATTAC 10320

************************************************************

LINE_R2_526 AAGTACAAACCAAACACAATATTCGAGAACAATAACCACAGACTTTACTGGGACAGAACC 3898

LOC110375617 AAGTACAAACCAAACACAATATTCGAGAACAATAACCACAGACTTTACTGGGACAGAACC 10380

************************************************************

LINE_R2_526 ATCATCACTGACAAAACCATACATTTTAATAGACCTGACATCACATTATACGATAAAATC 3958

LOC110375617 ATCATCACTGACAAAACCATACATTTTAATAGACCTGACATCACATTATACGATAAAATC 10440

************************************************************

LINE_R2_526 AATAGAATTGCCTACCTTATCGACATAGCCATCCCAAATACACATAACGTACAATCTACA 4018

LOC110375617 AATAGAATTGCCTACCTTATCGACATAGCCATCCCAAATACACATAACGTACAATCTACA 10500

************************************************************

LINE_R2_526 ATATCTGATAAACTGACCAAATACCAAGATCTAGCAATAGAATTAAAACGCCAATGGCAA 4078

LOC110375617 ATATCTGATAAACTGACCAAATACCAAGATCTAGCAATAGAATTAAAACGCCAATGGCAA 10560

************************************************************

LINE_R2_526 GCGCTANNNNNNNNNNNNNNNNNNNNNNNNNNNNNNNNNNNNNNNNNNNNNNNNNNNNNN 4138

LOC110375617 GCGCTANNNNNNNNNNNNNNNNNNNNNNNNNNNNNNNNNNNNNNNNNNNNNNNNNNNNNN 10620

************************************************************

LINE_R2_526 NNNNNNNNNNNNNNNNNNNNNNNNNNNNNNNNNNNNNNNNNNNNNNNNNNNNNNNNNNNN 4198

LOC110375617 NNNNNNNNNNNNNNNNNNNNNNNNNNNNNNNNNNNNNNNNNNNNNNNNNNNNNNNNNNNN 10680

************************************************************

LINE_R2_526 NNNNNNNNNNNNGTTTTTCTTTCTTCCTATTTTGTTTATTTTGTGATTACACGTTGTTCA 4258

LOC110375617 NNNNNNNNNNNNGTTTTTCTTTCTTCCTATTTTGTTTATTTTGTGATTACACGTTGTTCA 10740

************************************************************

LINE_R2_526 CTAGGTATCAATTTATTTATTTTTGGCACAGTTTTAAGGTATAGGTTTTCGTCTACAGAT 4318

LOC110375617 CTAGGTATCAATTTATTTATTTTTGGCACAGTTTTAAGGTATAGGTTTTCGTCTACAGAT 10800

************************************************************

LINE_R2_526 ATGTATATAGCACACACAAATTAATGCAGATTTAACCAACTTTACGAATTTATAAGCACA 4378

LOC110375617 ATGTATATAGCACACACAAATTAATGCAGATTTAACCAACTTTACGAATTTATAAGCACA 10860

************************************************************

LINE_R2_526 AGCACACGGAAACAACCGTCTGACTCACACGGGCGCGCGCGGGGAACCATATTGAACCCT 4438

LOC110375617 AGCACACGGAAACAACCGTCTGACTCACACGGGCGCGCGCGGGGAACCATATTGAACCCT 10920

************************************************************

LINE_R2_526 ACACTTTAAAGACAAAGTTTAAAATTGATTACAGAAAGACAGGTTGTAGCAGGTTGTTGT 4498

LOC110375617 ACACTTTAAAGACAAAGTTTAAAATTGATTACAGAAAGACAGGTTGTAGCAGGTTGTTGT 10980

************************************************************

LINE_R2_526 TCTACCGTATACATATATTTAAATTAATTAACTTTACTTCTTACTCCACAGCATGTACCA 4558

LOC110375617 TCTACCGTATACATATATTTAAATTAATTAACTTTACTTCTTACTCCACAGCATGTACCA 11040

************************************************************

LINE_R2_526 GCTAATCTTCGAAAGGATGACAAAATGGTGGCTGCAAATACCCCTCATCTACTGGCTGGG 4618

LOC110375617 GCTAATCTTCGAAAGGATGACAAAATGGTGGCTGCAAATACCCCTCATCTACTGGCTGGG 11100

************************************************************

LINE_R2_526 GGGGAAGAAGAAGAAGCAACTATACTTCCAAAAGCTGGTCGATGATTTCTCCAGCGATAT 4678

LOC110375617 GGGGAAGAAGAAGAAGCAACTATACTTCCAAAAGCTGGTCGATGATTTCTCCAGCGATAT 11160

************************************************************

LINE_R2_526 CGTGAGAAGAAGAAGAAAGGCCCTAGAGGTTTCTACACCTGATGAAGAATGTATGGGAGT 4738

LOC110375617 CGTGAGAAGAAGAAGAAAGGCCCTAGAGGTTTCTACACCTGATGAAGAATGTATGGGAGT 11220

************************************************************

LINE_R2_526 TGCAGATCGATATATATTGCAAGGGGATTTGACTGAAAATGAAATTAAGAGGGATACCTT 4798

LOC110375617 TGCAGATCGATATATATTGCAAGGGGATTTGACTGAAAATGAAATTAAGAGGGATACCTT 11280

************************************************************

LINE_R2_526 TACTTTATTTACTACGGTGAGGATTTTTTATGTTTAAGAATAATGTAAAACATACTTATG 4858

LOC110375617 TACTTTATTTACTACGGTGAGGATTTTTTATGTTTAAGAATAATGTAAAACATACTTATG 11340

************************************************************

LINE_R2_526 GGGCTAGTGGGGCTAAGTATCAGACGTGGGGATCGATCATGAGATCCTGGAACGCTGAGC 4918

LOC110375617 GGGCTAGTGGGGCTAAGTATCAGACGTGGGGATCGATCATGAGATCCTGGAACGCTGAGC 11400

************************************************************

LINE_R2_526 GCGGTCGATTCTTGTCTGATAATTTCCTCGATGTTTCGGAAAATTGACCCTAAACATGAG 4978

LOC110375617 GCGGTCGATTCTTGTCTGATAATTTCCTCGATGTTTCGGAAAATTGACCCTAAACATGAG 11460

************************************************************

LINE_R2_526 ACTCAGAAACAAGTTAATCTGGCAACCAGTCCTAAAACAGTACTGATAGACTGTTGCTAC 5038

LOC110375617 ACTCAGAAACAAGTTAATCTGGCAACCAGTCCTAAAACAGTACTGATAGACTGTTGCTAC 11520

************************************************************

LINE_R2_526 AGGTAAAACTCTGGTATTCAGCTGCATTCGTTTAGAGTTTAGACTGAGAGCCAAACCCAA 5098

LOC110375617 AGGTAAAACTCTGGTATTCAGCTGCATTCGTTTAGAGTTTAGACTGAGAGCCAAACCCAA 11580

************************************************************

LINE_R2_526 CATAATTGGGAAAAGGTTAGGTAATTGATAAGATGATCAATTTAAATCAATTTCTACATA 5158

LOC110375617 CATAATTGGGAAAAGGTTAGGTAATTGATAAGATGATCAATTTAAATCAATTTCTACATA 11640

************************************************************

LINE_R2_526 TGCTATCTCTAGAGTGTAGTAGAACATGTGTACTATTCAACGTAGATATTATTACTTTTC 5218

LOC110375617 TGCTATCTCTAGAGTGTAGTAGAACATGTGTACTATTCAACGTAGATATTATTACTTTTC 11700

************************************************************

LINE_R2_526 AGAGCCAAGAAGCAGCAGCAAAAATCGCATCAGGAGTTCTTATGTTCCTCGCCCATTTAC 5278

LOC110375617 AGAGCCAAGAAGCAGCAGCAAAAATCGCATCAGGAGTTCTTATGTTCCTCGCCCATTTAC 11760

************************************************************

LINE_R2_526 CGGATTGGCAGGTA---------------------------------------------- 5292

LOC110375617 CGGATTGGCAGGTACTAACATCTTATATTGGGATGTCACATGTCACATTTCTCGCTGAAG 11820

**************

LINE_R2_526 ------------------------------------------------------------ 5292

LOC110375617 AAAGATAAGTCTTAATTGCTGCTGTTTCCCATCCTCTCAACCGCATCCTGAGGAACTTCT 11880

LINE_R2_526 ------------------------------------------------------------ 5292

LOC110375617 TCGCGAATCAAGATATCAAGATAGCCTATGTTACCTAAGAAAAGTGTAGCTTTCACATCC 11940

[….]

**Figure S1. Alignment of LINE_R2526 element with Cytochrome P450 4g15-like gene**

LINE_RTE84107 ------------------------------------------------------------ 0

LINE_RTE17512 ------------------------------------------------------------ 0

LOC113006340 CAGACGGACAGACGGACAGACGGACAGACGGACAGACGGACATGACGAAACTATAAGGGT 60

LINE_RTE84107 ------------------------------------------------------------ 0

LINE_RTE17512 ------------------------------------------------------------ 0

LOC113006340 TCCGTTTTTGCCATTTTGGCTACGGAACCCTAAAAAGTTATTCTATATAAAGAGTGTACA 120

LINE_RTE84107 ------------------------------------------------------------ 0

LINE_RTE17512 ------------------------------------------------------------ 0

LOC113006340 AAATTCTTAAATTATTTGTTGAAGAGAAGAACGGAAAAATAAGACATGATAAATGCGAAC 180

[….]

LINE_RTE84107 ------------------------------------------------------------ 0

LINE_RTE17512 --------------ACCCGCAGAGCATTGTATGTTCTCTGAGTATAGTTAGCCCACAGAG 46

LOC113006340 CGTTATTAAATTGTACCCGCAGAGCATTGTATGTTCTCTGAGTATAGTTAGCCCACAGAG 5700

LINE_RTE84107 ------------------------------------------------------------ 0

LINE_RTE17512 CTCCCGTGTACAGTGATGTACAATATGCTTTAAATAATGTAATTTTAACTGCGTCTGTAC 106

LOC113006340 CTCCCGTGTACAGTGATGTACAATATGCTTTAAATAATGTAATTTTAACTGCGTCTGTAC 5760

LINE_RTE84107 ------------------------------------------------------------ 0

LINE_RTE17512 AACGGGAGAATCTGCGGGCCAACATATTCGCCCGAACTGACAATGATCTGCGTTCTCTCT 166

LOC113006340 AACGGGAGAATCTGCGGGCCAACATATTCGCCCGAACTGACAATGATCTGCGTTCTCTCT 5820

LINE_RTE84107 ------------------------------------------------------------ 0

LINE_RTE17512 CCATATCAACATCATCTTTTAAATCATCGGTGACCCAATGTCCAAGGTATTTAAATTGGT 226

LOC113006340 CCATATCAACATCATCTTTTAAATCATCGGTGACCCAATGTCCAAGGTATTTAAATTGGT 5880

LINE_RTE84107 ------------------------------------------------------------ 0

LINE_RTE17512 ACACTCTAGATAACGCTTCACCGTTCAGGTGAAGTGGCTGTACTGGTGGACTTTTGGGAC 286

LOC113006340 ACACTCTAGATAACGCTTCACCGTTCAGGTGAAGTGGTTGTACTGGTGGACTTTTGGGAC 5940

LINE_RTE84107 ------------------------------------------------------------ 0

LINE_RTE17512 CCGATTTAAAAACCATGTATACAGTCTTTTTCACGTTATATATAAGTCCGTGTGAAGCCA 346

LOC113006340 CCGATTTAAAAACCATGTATACAGTCTTTTTCACGTTATATATAAGTCCGTGTGAAGCCA 6000

LINE_RTE84107 ------------------------------------------------------------ 0

LINE_RTE17512 CAAAAGACTCACAAGCTGCGAGCAACTTCCGGAGAGCTCCGATTGAGGGGGCCAGCAGCA 406

LOC113006340 CAAAAGACTCACAAGCTGCGAGCAACTTCCGGAGAGCTCCGATTGAGGGGGCCAGCAGCA 6060

LINE_RTE84107 ------------------------------------------------------------ 0

LINE_RTE17512 CCATGTCGTCCGCATAGCTGAAGTTGTTACAACAAACACCATCAACATAGCAGCCGACAT 466

LOC113006340 CCATGTCGTCCGCATAGCTGAAGTTGTTACAACAAACACCATCAACATAGCAGCCGACAT 6120

LINE_RTE84107 ------------------------------------------------------------ 0

LINE_RTE17512 GCATGCCGCTGAGCTCCTCAACCAGGGCGTTTACGTACAGGTTGAAGAGTAGGGGTGAGC 526

LOC113006340 GCATGCCGCTGAGCTCCTCAACCAGGGCGTTTACGTACAGGTTGAAGAGTAGGGGTGAGC 6180

LINE_RTE84107 ------------------------------------------------------------ 0

LINE_RTE17512 TCAAACCACCCTGTCGCACCCCACACTCCAACCCACTCGCATCCGAAAGTTCACCCGCCC 586

LOC113006340 TCAAACCACCCTGTCGCACCCCACACTCCAACCTACTCGCATCCGAAAGTTCACCCGCCC 6240

LINE_RTE84107 ------------------------------------------------------------ 0

LINE_RTE17512 ACTTCACACGGTTCTCTTGGTTGAGATACCAGTA-------------------------- 620

LOC113006340 ACTTCACACGGTTCTCTTGGTTGAGATACCAGTACCTCAGGATTGATGCCAACTCTGGTG 6300

LINE_RTE84107 ------------------------------------------------------------ 0

LINE_RTE17512 ------------------------------------------------------------ 620

LOC113006340 GCACTCCAGTATCTGCTAACTTCTGCCACAGAACATCGTATGAGACTAAATCAAATGCCT 6360

[….]

LINE_RTE84107 ------------------------------------------------------------ 0

LINE_RTE17512 ------------------------------------------------------------ 620

LOC113006340 CTATTATTCGTACATTTCCAAAAATTTTTAAAATCGGACTTTGAATGGTGCTTAGCTAAG 7020

LINE_RTE84107 ATATCCATCTTGATTTGTTCCTGGTGGTTCTGACACCACTTTAATTTACTTTTAAATTCT 60

LINE_RTE17512 ------------------------------------------------------------ 620

LOC113006340 ATATCCATCTTGATTTGTTCCTGGTGGTTCTGACACCACTTTAATTTACTTTTAAATTCC 7080

LINE_RTE84107 TTACGTGCCTGTGTCATTTCAGTAAACGTATCCGAGTTACTTGGTTTACCACTAGATGCC 120

LINE_RTE17512 ------------------------------------------------------------ 620

LOC113006340 TTACGTGCCTGTGACATATCAATAAATGTTTCAGAGTTACTTGGTTTACCACTAGATGCC 7140

LINE_RTE84107 CATATGTTGAACTTTAGCCTAGCATTCCTGTAAGCCTCGCTTACATGTTTATTCCAACCT 180

LINE_RTE17512 ------------------------------------------------------------ 620

LOC113006340 CATATGTTAAACTTTAGCCTAGCATTCCTGTAAGCCTCGCTCACATGCTTATTCCAACCT 7200

LINE_RTE84107 GCCAGCAGCTTTTTACGTCTACCCTTATTTGTTACGTAAGAGAAACGTGCAGCGTCACAT 240

LINE_RTE17512 ------------------------------------------------------------ 620

LOC113006340 GCCAGCAGCTTCTTACGTCTACTCTTATTTGTTACGTAAGAGAAACGTGCAGCGTCACAT 7260

LINE_RTE84107 AAAATATTTACTACATTTATATACATTCTATCTATAATTTGCTTATGACTATATAAATTA 300

LINE_RTE17512 ------------------------------------------------------------ 620

LOC113006340 AAAATACTTACTACATTTATATACATTTTATCTATAATTTGCTTATGACTATATAAATTA 7320

LINE_RTE84107 CAATGTTTACCACTACAAGATTGAAACTCTATCGGGAAATCTACATATTTTAACTTAGCA 360

LINE_RTE17512 ------------------------------------------------------------ 620

LOC113006340 CAATGTTTACCACTACAAGATTGAAACTCTATAGGGAAATCTACATATTTTAACTTAGAA 7380

LINE_RTE84107 TTACATATATTTTTAAATTTATTAATTTGAGATCCATTTCTATTACCCCAGTGTACTTTA 420

LINE_RTE17512 ------------------------------------------------------------ 620

LOC113006340 TTACATAAATTATGATATTTATTAATTTGAGATCCATTTCTATTACCCCAGTGTACTCTA 7440

LINE_RTE84107 TTACATGGTGATGTTTTTAATTGAGTTTTATGCAATACTATAT-----TTAAATTACACT 475

LINE_RTE17512 ------------------------------------------------------------ 620

LOC113006340 TTACATGGTGATGTTTTTAATTGAGTTTTATGCAATACTATATTTTATTTAAATTACACT 7500

LINE_RTE84107 TTATAACTAGTGGGTAGTGATCAGACCAATATATGTCATGTTTCACATATATATATCTGT 535

LINE_RTE17512 ------------------------------------------------------------ 620

LOC113006340 TAATAACTAGTGGGTAGTGATCAGACCAATATATGTCATGTTTAACATA--TATATCTGT 7558

LINE_RTE84107 AATACAGATAGCAGCTGCCTGAGTAACAATACAGTGGTCTAACCACCTTTTACTATTATG 595

LINE_RTE17512 ------------------------------------------------------------ 620

LOC113006340 AATACAAATGGCAGCTGCCTGAGTAACAATACAGTGGTCTAACCACCTTTTACTGTTATG 7618

LINE_RTE84107 TGCTTCACTAATGAAAGTAAAAGTACCTTGCAAATTGTGATGATTTAGATCCGCACATGT 655

LINE_RTE17512 ------------------------------------------------------------ 620

LOC113006340 TGCTTCACTAATGAATGTAAAAGTACCTTGCAAATTCTGGTGATTTAGATCCGCACAAAT 7678

LINE_RTE84107 CCACCTTTGCTCGCGTGAAAAGCTTAGGAGCTCATCACAAAAACGTTCACCGGGATGTGC 715

LINE_RTE17512 ------------------------------------------------------------ 620

LOC113006340 CCACCTTTGCTCACGTGAAAAGCTTAGGAGCTCATCACAAAAACGTTCACCGGGATGTGC 7738

LINE_RTE84107 GTTAAAATCCCCCAATACAAATACT----------------------------------- 740

LINE_RTE17512 ------------------------------------------------------------ 620

LOC113006340 GTTAAAATCCCCCAATACAAATACTGACTCTACATTGCTGTCCTGTATGATTGCACTAAT 7798

LINE_RTE84107 ------------------------------------------------------------ 740

LINE_RTE17512 ------------------------------------------------------------ 620

LOC113006340 TGCGCCTAAACAGTCCGTAAACTCGGGTAAGTTCTCACTGCAGTCAACGGGCATATAAAC 7858

[….]

**Figure S2. Alignment of LINE_RTE84107 and LINE_RTE17512 elements with Cytochrome P450 4C-like gene**

TIR_CACTA3565 ------------------------------------------------------------ 0

LOC110381376 TCAGACATGGTGTTGTTAGTACTGGCCATAGCTTGTTGCGTGTTGTTCGCTGTATGGAGA 60

TIR_CACTA3565 ------------------------------------------------------------ 0

LOC110381376 CTGGTGAAGCCGAGGTCTCCATCATCTCCCGAAAGCCCTGAGAAGATAGCTGACTCTTTT 120

TIR_CACTA3565 ------------------------------------------------------------ 0

LOC110381376 TGTAAGACATTCACTTCATTATTTGTTTAACTTCGTTTTTAATCATATCAAGATGTACCT 180

TIR_CACTA3565 ------------------------------------------------------------ 0

LOC110381376 ATTTTCAAATTACAACCACACTGGCCTTATTTTCGGCCGTATGGACACATTTCTTCTTTA 240

[….]

TIR_CACTA3565 ----------------------------------------------------------TA 2

LOC110381376 TGTCCTCTTAAAACCGGTGGCTGGCCATTTCATAAGTTTGGAGTTGAGGGTGTAATAATA 6900

**

TIR_CACTA3565 ACACTAGTTTACACGGTTTTTATACCCCAGATGAAAGTTGTTGAATTTTATCTATCTATC 62

LOC110381376 ACACTAGTTTACACGTTTTTTATACCCCAGATGAAAGTTGTTGAATTTTATCTATCTATC 6960

*************** ********************************************

TIR_CACTA3565 TATAGGTACTCTAGGAAAAATATTTAAAAAAAATCTATCACGTCCACATTTGGAATTATA 122

LOC110381376 TATAGGTACTCTAGGAAAAATATTTAA-AAAAATCTATCACGTCCACATTTGGAATTATA 7019

*************************** ********************************

TIR_CACTA3565 TTTAATTTAAAAAACACGAGAAAACGGACCATAAACAACTAAAAATTATTAATTTTGGAC 182

LOC110381376 CTTAATTTAAAAAACACGAGAAAACGGACCATAAGCAACTAAAAATTATTAATTTTGGAC 7079

********************************* *************************

TIR_CACTA3565 TTCTTTTTGTAGGTTTCGGAATCGGTTTCTCTTAATATAGACCAGGAAGTAGTCTCCCAA 242

LOC110381376 TTCTTTTTGTAGGTTTAGGAATCGGTTTCTTTTAATATAGACCAG-CAGTAGTCTCCCAA 7138

**************** ************* ************** *************

TIR_CACTA3565 CATATTTTCGTCCCAATAGCCTTGATGACGATCTTCAAAAGCCTTTAAATCCTGGTGAAA 302

LOC110381376 CATATTTTCGTCCTAATAGCCTTGATGACGATCTTCAAAAGCCTTTAAATCCTGGTGAAA 7198

************* **********************************************

TIR_CACTA3565 ATGCTCTCCTTGTTCGTCGCTCATGCTTCCTAAGTTTTCCGGAAAAAAATCCAAGTGGGA 362

LOC110381376 ATGCTCTCCTTGTTCGTCTCTGATGCTTCCTAAGTTTTCCGGAAAAAAATCCAAGTGGGA 7258

****************** ** **************************************

TIR_CACTA3565 ATGTAGAAAATGAATCTTTTGACATGTTAACACCCATCTTATGATAATTTTGTAATGAAT 422

LOC110381376 ATGTAGAAAATTAATCTTTTGACATGTTAACACCCATCTTATGATAATTTTGTAATGAAT 7318

*********** ************************************************

TIR_CACTA3565 CACTCACTATCTCTTTATTGTTTTCACTTTTATTATTGCTTTAAAAAGTCTCTAACAACA 482

LOC110381376 CACTCACGATCTCTATTGTTTT--CACTTTT-ATTATCGCTTAAAAGGTCTCTAACAACA 7375

******* ****** * * ** ******* * * ****** *************

TIR_CACTA3565 GTTTTGAATGAATTCCAAGCAACTCTTTCAGTCGTAGATAAAAGAGTATCAAACTTAAAA 542

LOC110381376 GTTTTGAATGAATTCCAAGCAACTCTTTCAGTCGTAAATAAAAGAGTATCAAACTTAAAA 7435

************************************ ***********************

TIR_CACTA3565 TCACTTATGATTTTGCGTACTTGTGGGACGCCAAACACCCCCTTTTTAA-----GTGACA 597

LOC110381376 TCACTTATGATTTTGCGTACTTGTGGGACGCCAAACACAGTGGCCCATCTTTAAATGACA 7495

************************************** *****

TIR_CACTA3565 TATGCATATGAAACAATACCCGACTTAGTTTTCCCTATTAAAACCCGAAGTCTTAGTAAC 657

LOC110381376 TATGCATAT----ACAATACCCGACTTAGTTTTCCTATTAAAACCCGAAGTCTTAGTGAC 7551

********* * ** * *** ************************ **

TIR_CACTA3565 ACAAAAGTAACAGTCATTTTCGTGATTAGTTGGTTCCCTGCACAACATTGGTGTACCAAA 717

LOC110381376 ACAAAATTAACAGTCATTTTCGTGATTAGTTGGTTCCCTGCACAACATTGGTGTACCAAA 7611

****** *****************************************************

TIR_CACTA3565 TGACAGGTGTACGTTTTCGCCGGATGACCATCACAGTAAAGTGATTCTACAGCTCTCACA 777

LOC110381376 TGACAGGTAAACGTTTTCGCCAGATGTCCATCACAGTAAAGAGATTCTACAGCTCTCACA 7671

******** *********** **** ************** ******************

TIR_CACTA3565 AAACACATAGTGAGTCCAGTTTTTTTCTTGATTGGCCACAGGAAATCCGAAATAATGAAG 837

LOC110381376 AAACACATAGTAGGCCCAGTTT-TTTCTTTATTGGCCACAGGAAATCCGAAATAATGAAG 7730

*********** * ******* ****** ******************************

TIR_CACTA3565 ATATGCAATTTTCAACGATTCTGTCATATGTCTTTTCTTTTTTATAAATATAAATTGTCC 897

LOC110381376 ATATGCAATTTTCAACGATTCTGTCATGT--CTTTTCTTCTTTATAAATATAAATTGTCC 7788

*************************** * ******** ********************

TIR_CACTA3565 ACAAACATAACAAAAGCGATCACAACTTTGTTTACACTGTCTACGCGACATTTTATTTGA 957

LOC110381376 ACAAACATAACAAAATCGATCACAACTTTGTTTACACTGTCTACGCGACATTTTTTTTAA 7848

*************** ************************************** *** *

TIR_CACTA3565 ATTAAACAAAAAATATCTCCTAAACTAAACAAGTAATGCAACACCAAAGATCGAGTAGTT 1017

LOC110381376 ATTAAACAAAAAATATCTCCTAAACTAAACAAGTAATGCAACACCAAAGATCGAGAGT-T 7907

******************************************************* *

TIR_CACTA3565 AAGAATGATTCCTAAAAACAAATATTTGAGGGTAGAAAAAAAAATGTGACGTGATAGAAT 1077

LOC110381376 AAGAATGATTCCTAAAAACAAATATTTGAGGGTAGAA-AAAAAATTTGGCGTGATAGAAT 7966

************************************* ******* ** ***********

TIR_CACTA3565 TTTTATGTTGTTACTTTTAGAATGTACAAAGGTGGCGGAGAGTACTGGTATAGATTTTGT 1137

LOC110381376 TTTTATGTTGTTACTTTTAGAAAGTACAAAGGTGGCAGAGAGTACTGGTATAGATTTTGT 8026

********************** ************* ***********************

TIR_CACTA3565 GAGGGGTATATAAAAAAAATAAAAATTTGTAAACTAGTGTTA------------------ 1179

LOC110381376 GAGGGGTATATGAAAAAAATAAAAAATTGTAAACTAGTGTAATAATTGTCAGTGGGCGGA 8086

*********** ************* ************** *

TIR_CACTA3565 ------------------------------------------------------------ 1179

LOC110381376 CTGAAGGATTGCCACAAAAGGTTTTACAGACTATTGGTTGCCTTATAGTTTCAAAAGATT 8146

TIR_CACTA3565 ------------------------------------------------------------ 1179

LOC110381376 TTTTAGTTCATTAATGATCGGAATTACCATCCTATTGCCACCCGCTTTTTAAATAAAGCA 8206

TIR_CACTA3565 ------------------------------------------------------------ 1179

LOC110381376 AGGCTATCCATAAATGGGCTATTTAAACATCATTTTCTATTTACATTTATATCTACATAC 8266

TIR_CACTA3565 ------------------------------------------------------------ 1179

LOC110381376 ATACATCTACATAACAGTAAACTGTCAACGTAAACTGACATTTCCATAATATACCTATTA 8326

TIR_CACTA3565 ------ 1179

LOC110381376 AGTACA 8332

**Figure S3. Alignment of TIR_CACTA3565 element with Cytochrome P450 4C-like gene**

LINE_Jockey2199 ------------------------------------------------------------ 0

LOC110381042 AAATTGTCTATCATTCGACAAGCTTTAGACAGCTGAAGTTTCTTACGGGACACCAATACT 60

LINE_Jockey2199 ------------------------------------------------------------ 0

LOC110381042 TCTTCTTGGTCCAAGGTTTTATTTTTGTCATTCTAAAATTGATCTTATTCCTAATCGTTT 120

LINE_Jockey2199 ------------------------------------------------------------ 0

LOC110381042 TTTGGTCTAATTTCCTTCAGAGCTTCATCAGCGTTTTTCTGGTCATCTTCACGACTGTTG 180

[….]

LINE_Jockey2199 CCATAAGGGCGGGGACAGAGACGATGTCAATAACTACAGACCCATATCAGTTTTGCCATC 680

LOC110381042 NNNNNNNNNNGGGGACAGAGACGATGTCAATAACTACAGACCCATATCAGTTTTGCCATC 9480

**************************************************

LINE_Jockey2199 AATATCTAAAGTAATAGAAAAGCTAATAAATAATAGACTATTAAAATATCTAAATAAATA 740

LOC110381042 AATATCTAAAGTAATAGAAAAGCTAATAAATAATAGACTATTAAAATATCTAAATAAATA 9540

************************************************************

LINE_Jockey2199 TGACTTACTTTCATCGACTCAATTTGGATTCAGGCAGGGTATTTCTACTGAGGATGCAGT 800

LOC110381042 TGACTTACTTTCATCGACTCAATTTGGATTCAGGCAGGGTATTTCTACTGAGGATGCAGT 9600

************************************************************

LINE_Jockey2199 AACCGCCCTAACTTCAGTAGTAACTGATGAGCTTGATAATGGAAATAAATGTGTTACAGT 860

LOC110381042 AACCGCCCTAACTTCAGTAGTAACTGATGAGCTTGATAATGGAAATAAATGTGTTACAGT 9660

************************************************************

LINE_Jockey2199 ATTCCTAGACTTAAAAAAAGCGTTTGACACCGTCTCTGTCCCAACCCTGGTAAACAAATT 920

LOC110381042 ATTCCTAGACTTAAAAAAAGCGTTTGACACCGTCTCTGTCCCAACCCTGGTAAACAAATT 9720

************************************************************

LINE_Jockey2199 GGAAAATATGGGAAT---------TAGAGGAATGCCACTTAATCTCTTAAAAAGTTATCT 971

LOC110381042 GGAAAATATGGGAATTAGAGGAATTAGAGGAATGCCACTTAATCTCTTAAAAAGTTATCT 9780

*************** ************************************

LINE_Jockey2199 TTCAAAAAGGAAACAGAAGATTAGAATAGGAGAGTTCACCAGTGAAGACGCAGAGGTATC 1031

LOC110381042 TTCAAAAAGGAAACAGAAGATTAGAATAGGAGAGTTCACCAGTGAAGACGCAGAGGTATC 9840

************************************************************

LINE_Jockey2199 ATACGGCGTACCGCAAGGAAGCGTATTAGGCCCTACACTATTCTTAACGTATATAAATGA 1091

LOC110381042 ATACGGCGTACCGCAAGGAAGCGTATTAGGCCCTACACTATTCTTAACGTATATAAATGA 9900

************************************************************

LINE_Jockey2199 TCTTTCTAGCTTAAAAATCAGAAATGCAAAAATTTTCTCATACGCCGATGATACCGCTGT 1151

LOC110381042 TCTTTCTAGCTTAAAATTCAGAAATGCTAAAATTTTCTCATACGCCGATGATACCGCTGT 9960

**************** ********** ********************************

LINE_Jockey2199 TGTCTTCTCAGGGAAATCCTGGCAAGAAGTTGAATATGCGGCTGAGGTGGGCATGGCTAA 1211

LOC110381042 TGTCTTCTCAGGGAAATCCTGGCAAGAAGTTGAATATGCGGCTGAGGCGGGCATGGCTAA 10020

*********************************************** ************

LINE_Jockey2199 AATAGCTAAATGGCTAATAAATAACCTACTCACATTAAACACTTCCAAAACTAATTTTAT 1271

LOC110381042 AATAGCTAAATGGCTAATAAATAACCTACTCACATTAAACACTTCCAAAACTAATTTTAT 10080

************************************************************

LINE_Jockey2199 ATGCTTCAGCATTAGTAATCGGACACAGCCTAGTGAGAATTTTAAAATTAAAATCCATCA 1331

LOC110381042 ATGCTTCAGCATTAGTAATCGGACACAGCCTAGTGAGAATTTTAAAATTAAAATCCATCA 10140

************************************************************

LINE_Jockey2199 CTGTGACAACATAAAAAGCCGAAATTGTGACTGTCCAAATATAGCCAAAGTAACACAAGC 1391

LOC110381042 CTGTGACAACATAAAAAGCCGAAATTGTGACTGTCCAAATATAGCCAAAGTAACACAAGC 10200

************************************************************

LINE_Jockey2199 CAAATACTTAGGTGTACTGGTAGACCAAAGACTATCTTGGTACCCCCACCTTGAACACGT 1451

LOC110381042 CAAATACTTAGGTGTACTGGTAGACCAAAGACTATCTTGGTACCCCCACCTTGAACACGT 10260

************************************************************

LINE_Jockey2199 AGCTTGTAGGGTTAGAAAATTGGGCTGGTTATTCAGCGTACTAAGACACGTTGTACCAAG 1511

LOC110381042 AGCTTGTAGGGTTAGAAAATTGGGCTGGTTATTCAGCGTACTAAGACACGTTGTACCAAG 10320

************************************************************

LINE_Jockey2199 GAAAGTGACTAGTCGCCATAACTCATTTAGAAATGTGTTAAATGAGATATATGTCTCTTT 1571

LOC110381042 GAAAGTGACTAGTCACCATAACTCATTTAGAAATGTGCTAAATGAGATATATGTCTCTTT 10380

************** ********************** **********************

LINE_Jockey2199 GGTGCAATCTGTCTTGGTGTACTGCATTCCTATTTGGGGCGGGTCTGCAAAAACTAAA-- 1629

LOC110381042 GGTGCAATCTGTCTTGGTGTACTGCATTCCTATTTGGGGCGGGTCTGCAAAAAGTAAATT 10440

***************************************************** ****

LINE_Jockey2199 ------------------------------------------------------------ 1629

LOC110381042 CATTGAGGTGGAGCGAGCTCAACGCGCTCTTATAAAAACAATGTATTTTAAAAAACGAAG 10500

LINE_Jockey2199 ------------------------------------------------------------ 1629

LOC110381042 GTACCCTACAGAAAATCTCTATCAGATTAGTTATCTACTCTCGGTAAGAAAATTATATAT 10560

LINE_Jockey2199 ------------------------------------------------------------ 1629

LOC110381042 TATACAAAGCATATTAAAAACACATAAGACCCTACCTTATGATGCATCCAAATTAAATAA 10620

[….]

**Figure S4. Alignment of LINE_Jockey2199 element with Cytochrome P450 6k1-like gene**

TIR_Mariner2770 ------------------------------------------------------------ 0

LOC110371343 GAAGCGGTTCGATCGTCTCTGCGAATAAGGGAGCTTGGATTTTCATTTGAACAAAATCTA 60

TIR_Mariner2770 ------------------------------------------------------------ 0

LOC110371343 AAAGATACTTTATTACTATGTCCAATGTGTTCAGAATGCTGGTGGGGCTGTCACGTTTTT 120

TIR_Mariner2770 ------------------------------------------------------------ 0

LOC110371343 GCAGTGCTTCGGGAATGTTCGCTGAGGCGAGGAAAGTTGGTATGAAATCCTCATTAAATT 180

[….]

TIR_Mariner2770 ------------------------------------------------------------ 0

LOC110371343 GTGAATCCTCAACACACTGTGCCGCTTTTAAAAGATGATGACTTTTACATTTGGGACAGG 5640

TIR_Mariner2770 -----------------ATACGAGGGCGGGTCAATAAGTCCGTGACTTTTTGAATTTCTG 43

LOC110371343 TATGTATAGATAGACATATACGAGGGCGGGTCAATAAGTCCGTGACTTTTTGAATTTCTG 5700

*******************************************

TIR_Mariner2770 ACCTCTTTACTGAAAAAGCAACACTACTCCTGTTAACAGGCATCTATCAGGTGACTCCTG 103

LOC110371343 ACCTCTTTACTGAAAAAGCAACACTACTCCTGTTAACAGGCATCTATCAGGTGACTCCTG 5760

************************************************************

TIR_Mariner2770 ACAAAATTTGAACTTGCTGCGTCAGTTAGATTGTGTTTGACAACTATCTTTATCAGACTA 163

LOC110371343 ACAAAATTTGAACTTGCTGCGTCAGTTAGATTGTGTTTGACAGCTATCTTTATCAGACTA 5820

****************************************** *****************

TIR_Mariner2770 CCCAGTAATCTGAGGAAAAAATGGAACAAAGTGAATTTCGTGTGCTCATTAAGCATTATT 223

LOC110371343 CCCAGTAATCTGAGGAAAAAATGGAACAAAGTGAATTTCGTGTGCTCATTAAGCATTATT 5880

************************************************************

TIR_Mariner2770 TTTTGCGAAAAAAAACCATTACCGAAACCAAGGCTAAGCTTGATAAATACTATGGGGACT 283

LOC110371343 TTTTGCGAAAAAAAACCATTACCGAAACCAAGGCTAAGCTTGATAAATACTATGGGGACT 5940

************************************************************

TIR_Mariner2770 CTGCACCATTGATTTCAATCGTAAAAAAGTGGTTTACTGAATTTCGTTGTGGCCGTACAA 343

LOC110371343 CTGCACCATTGATTTCAATCGTAAA-AAGTGGTTTACTCAATTTCGTTGTGGCCGTACAA 5999

************************* ************ *********************

TIR_Mariner2770 GCACTGAAGATGCCGAACGCCCTGGACGCCCAGTTGAAGTCTCTTCACCCGAAACAATCG 403

LOC110371343 GCACTGAAGATGCCGAACGCCCTGGACGCCCAGTTGAAGTCTCTTCACCCGAAACAATCG 6059

************************************************************

TIR_Mariner2770 AAAAAATCCACGGTATGGTGTTGGCCGACCGAAGATTGAAAGTGCAAGAGATTGTGGAAG 463

LOC110371343 AAAAAATCCACGGTATGGTGTTGGCCGACCGAAGATTGAAAGTGCAAGAGATTGTGGAAG 6119

************************************************************

TIR_Mariner2770 CCGTAGGGATCTCGCATGGCTCAGTGGTTTCAATTTTGAATGATCACTTGGGCATGAGAA 523

LOC110371343 CCGTAGGGATCTCGCATGGCTCAGAGGTTTCAATTTTGAATGATCACTTGGGCATGAGAA 6179

************************ ***********************************

TIR_Mariner2770 AGCTTTCCGCAAGATGGGTGCCGCGTTTGCTCACAGTCGACCACAAACGCAATCGTGTAA 583

LOC110371343 AGCTTTCCGCAAGATGGGTGCCGCGTTTGCTCACAGTCGACCACAAACGCAATCGTGTAA 6239

************************************************************

TIR_Mariner2770 CAACTTCACAGGAAGGTTTGGCGTTGTTTGATCGCAATAAGGAGGAGTTTTTGCGCCGTT 643

LOC110371343 CAACTTCACAGGAAGGTTTGGCGTTGTTTGATCGCAATATGGAGGAGTTTTTGCGCCGTT 6299

*************************************** ********************

TIR_Mariner2770 TTGTAACAGTGGACGAAACATGGATCCATCACAACACACCAGAGACCAAACAACAGTCAA 703

LOC110371343 TTGTAACAGTGGACGAAACATGGATCCATCACAACACACCAAAGACCAAACAACAGTCAA 6359

***************************************** ******************

TIR_Mariner2770 AACAGTGGGTTTCTAAGGGTGAATCGGCACCAAAGAAGGCCAAGGTGAGTTTGTCAGCCA 763

LOC110371343 AACAGTGGGTTTCTAAGGGTGAATCGGCACCAAAGAAGGCCAAGGTGAGTTTGTCAGCCA 6419

************************************************************

TIR_Mariner2770 ATAAAGTTATGGCGACTGTTTTTTGGGATGCACGCGGGATAATCCACATTGACTACCTTC 823

LOC110371343 ATAAAGTTATGGCGACTGTTTTTTGGGATGCACGCGGGATAATCCACATTGACTACCTTC 6479

************************************************************

TIR_Mariner2770 AAAAGGGAAGAACAATCAATGGGGAATATTATGCCACCTCATTACCTACCGCTTCAAATA 883

LOC110371343 AAAAGGGAAGAACAATCAATGGGGAATATTATGCCACCTCATTACCTACCGCTTCAAATA 6539

************************************************************

TIR_Mariner2770 CGATTTGAAAAAAAAACCGACCTCATTTGGCCAAGAAAAAAGTTCTTTTCCACCAAGACA 943

LOC110371343 CGATTTGAAAAAAAAACCGACCTCATTTGGCCAAGAAAAAAGTTCTTTTCCACCAAGACA 6599

************************************************************

TIR_Mariner2770 ATGCAAGCACACATGTGCAGTTGCCATGGCAAAAATCCATGAATTCGGCTATGAATTGCT 1003

LOC110371343 ATGCAAGTAAACATGTGCAGTTGCCATGGCAAAAATCCATGAATTGGGCTATGAATTGCT 6659

******* * *********************************** **************

TIR_Mariner2770 CCCTCATCCGCCCTATTCTCCAGATTTGGCTCCGAGTGACTACTTCTTGTTCCCAAACCT 1063

LOC110371343 CCCTCATCCGCCCTATTCTCCAGATTTGGCTCCGAGTGACTACTTCTTGTTCCCAAACCT 6719

************************************************************

TIR_Mariner2770 GAAGAAATGGCTTGGTGGAAAAAGATTTGACTCCAATGATGAAGTCATCTCGCAAACAAG 1123

LOC110371343 GAAGAAATGGCTTGGTGGAAAAATATTTGACTCCAATGATGAAGTCATCTCGCAAACAAG 6779

*********************** ************************************

TIR_Mariner2770 GGCCTATTTTGAGGACCTCGACAAATCCTAT------TTTTTGGAAGGGATAAAAAAATT 1177

LOC110371343 GGCCTATTTTGAGGACCTCGACAAATCCAATCAAAAAAATTTGGAAGGGATAAAAAAATT 6839

**************************** ** *********************

TIR_Mariner2770 GGAGAAGCGTTGGACAAAGTGTATAGAGCTCAAGGGGGACTACGTTGAAAAATAAAATGA 1237

LOC110371343 GGAGAAGCGTTGGACAAAGTGTATAGAGCTCAAGGGGGACTACGTTAAAAAATAAAATGA 6899

********************************************** *************

TIR_Mariner2770 TTTATTTATCAAAAAATCTGTGTTTTATTCCAAAAGTCACGGACTTATTGACCCGCCCTC 1297

LOC110371343 TTTATTTATCAAAAAATCTGTGTTTTATTCCAAAAGTCACGGACTTATTGACCCGCCCTC 6959

************************************************************

TIR_Mariner2770 GTA--------------------------------------------------------- 1300

LOC110371343 GTATTTATCGTGTAATCGTGGTATAACGACAATTATTGTAATCTTTGCAGTCACGCCATT 7019

***

TIR_Mariner2770 ------------------------------------------------------------ 1300

LOC110371343 GCTATATATTTGGTGACCAAATATGGAGCCGACGATTCTCTATACCCAGCTGATCCGAAA 7079

TIR_Mariner2770 ------------------------------------------------------------ 1300

LOC110371343 AAGAGGGCCGTCATTGATCAACGTCTCCACTTTGATAGTGGGATTCTGTTTCCAGCCTTA 7139

[….]

**Figure S5. Alignment of TIR_mariner2770 element with glutathione S-transferase 1-like gene**

LINE_RTE63004 ------------------------------------------------------------ 0

LOC110377844 TCGCGCCGCGGTCGACCCGCGAACAGCGCATTTAAAAAACCTACTCGGTATCGCGCGGGA 60

LINE_RTE63004 ------------------------------------------------------------ 0

LOC110377844 AAATAGTGACAGTTCGATTTTTTAATTCCATAGTCAACGTCAGATATTATTTGTCAAAAC 120

LINE_RTE63004 ------------------------------------------------------------ 0

LOC110377844 TCGGACATTATGACAGATTGATTTTTTAATTTAATTAAATAGTCAAAGTCAGTTTATTTT 180

[….]

LINE_RTE63004 ------------------------------------------------------------ 0

LOC110377844 AACTTATGTATCCTAAAATACACCAATCCTTAGGTGGTAGGAGAGTGACCACCAACAATT 38580

LINE_RTE63004 ---------AAGAGGGTTGACGTCAGGCAGGCGGCCGGTCGTAAAAACACAATGCCAAAT 51

LOC110377844 GCATTCCCAAAGAGGGTTGACGTCAGGCAGGCGGCCGGTCGTAAAAACACAATGCCAAAT 38640

***************************************************

LINE_RTE63004 CATATAGCAATATGTCGAATGTTGAAGAGATGAGCGATAGGGCTAGGGCGTACCCCTCAG 111

LOC110377844 CATATAGCAATATGTCGAATGTTGAAGAGATGAGCGATAGGGCTAGGGCGTACCCCTCAG 38700

************************************************************

LINE_RTE63004 GCGACGCGCAGGGGCAGCACCCGGCCCCTGTGGGAAGTGGACAAGGGTTTTTGCACCAAC 171

LOC110377844 GCGACGCGCAGGGGCAGCACCCGGCCCCTGTGGGAAGTGGACAAGGGTTTTTGCACCAAC 38760

************************************************************

LINE_RTE63004 CTGGGCGGGTGCAATGTAAGAAGCGAGTCCGGGAAGTAAGATTGAGGTATGCAAGTTGGA 231

LOC110377844 CTGGGCGGGTGCAATGTAAGAAGCGAGTCCGGGAAGTAAGATTGAGGTATGCAAGTTGGA 38820

************************************************************

LINE_RTE63004 ATGTAGGAACGATGACTGGAAGAGCCAGAGAGTTAGCGGATGTATTAAAGAGAAGACGAA 291

LOC110377844 ATGTAGGAACGATGACTGGAAGAGCCAGAGAGTTAGCGGATGTATTAAAGAGAAGACGAA 38880

************************************************************

LINE_RTE63004 TAAATGTGGCATGTTTGCAAGAGACTAAATGGAAGGGCACGAAAGCTAGAGAAATTGGGG 351

LOC110377844 TAAATGTGGCATGTTTGCAAGAGACTAAATGGAAGGGCACGAAAGCTAGAGAAATTGGGG 38940

************************************************************

LINE_RTE63004 AAGGATACAAGTTTTATTATTGTGGAAGTGATGGGAAAAGGAATGGGGTAGGCATTGTGT 411

LOC110377844 AAGGATACAAGTTTTATTATTGTGGAAGTGATGGGAAAAGGAATGGGGTAGGCATTGTGT 39000

************************************************************

LINE_RTE63004 TAGATAAGAACTTGAAAAAAAGTGTGATAGATGTAAAGAGAGTGAATGATAGAATAATAG 471

LOC110377844 TAGATAAGAACTTGAAAAAAAGTGTGATAGATGTAAAGAGAGTGAATGATAGAATAATAG 39060

************************************************************

LINE_RTE63004 TTGTTAAAATAATGTATGAAAGTTTGATAATAAATGTTATAAGTGTGTATGCTCCTCAAG 531

LOC110377844 TTGTTAAAATAATGTATGAAAGTTTGATAATAAATGTTATAAGTGTGTATGCTCCTCAAG 39120

************************************************************

LINE_RTE63004 TCGGTTGTGATGACAGGGTGAAAGAACAATTTTGGATGGATTTCGATGCAGTAATGATGA 591

LOC110377844 TCGGTTGTGATGACAGGGTGAAAGAACAATTTTGGATGGATTTCGATGCAGTAATGATGA 39180

************************************************************

LINE_RTE63004 ATGTGCCGACGAATGAACAGGTATTTGTGGGAGGAGACTTTAATGGCCATGTTGGCAGAA 651

LOC110377844 ATGTGCCGACGAATGAACAGGTATTTGTGGGAGGAGACTTTAATGGCCATGTTGGCAGAA 39240

************************************************************

LINE_RTE63004 TGAGAGGGAATTACGAAAGAGTGCATGGAGGGTGGGGGTTCGGTTGCCAGAATGACGAGG 711

LOC110377844 TGAGAGGGAATTACGAAAGAGTGCATGGAGGGTGGGGGTTCGGTTGCCAGAATGACGAGG 39300

************************************************************

LINE_RTE63004 GTGAAGCCTTGCTACAGGCTGCTACTGCGTTTGACCTAGCAGTGGTAAATACGTGGTTTC 771

LOC110377844 GTGAAGCCTTGCTACAGGCTGCTACTGCGTTTGACCTAGCAGTGGTAAATACGTGGTTTC 39360

************************************************************

LINE_RTE63004 AAAAGAACATCGAACATCTGATCACCTATAAAAGCGGTCACCACGTGACACAAATAGATT 831

LOC110377844 AAAAGAACATCGAACATCTGATCACCTATAAAAGCGGTCACCACGTGACACAAATAGATT 39420

************************************************************

LINE_RTE63004 ACTTTTTGGTCAGACGCAGTAGTTTAAAGAACATCAAAGACTGCAAGGTGATACCAGGCG 891

LOC110377844 ACTTTTTGGTCAGACGCAGTAGTTTAAAGAACATCAAAGACTGCAAGGTGATACCAGGCG 39480

************************************************************

LINE_RTE63004 AAG--------------------------------------------------------- 894

LOC110377844 AAGCTTTAGTCTCGCAGCACCGACCCCTAATTATGGATGTGATTTTAACTTCCCGGCCAA 39540

***

LINE_RTE63004 ------------------------------------------------------------ 894

LOC110377844 AAGCCAAAGAGAGACGGGCCCCCAAAATTAAATGGCATCTGTTGGGGAAGGCTGAGTTGG 39600

LINE_RTE63004 ------------------------------------------------------------ 894

LOC110377844 CCCAGGAATTTAGAAAAGTAGTGGTTGATAAGATGATTGAAATGGGAGAAATGAATGAAA 39660

[….]

**Figure S6. Alignment of LINE_RTE63004 element with ABC-C1 homolog 49-like gene**

TIR_Mariner419 ------------------------------------------------------------ 0

TIR_hAT2824 ------------------------------------------------------------ 0

LOC110376033 CTTACCACAACCGGCGACGCCACTGTCCGTAACGTACGTGGCGACCGTAGTCACCGACTC 60

TIR_Mariner419 ------------------------------------------------------------ 0

TIR_hAT2824 ------------------------------------------------------------ 0

LOC110376033 GTGTACCAATTGTTTATGTTGTGTTCCGTTTTTTAATTCCGTTTAATTATTGAAAAAGAA 120

TIR_Mariner419 ------------------------------------------------------------ 0

TIR_hAT2824 ------------------------------------------------------------ 0

LOC110376033 AGTGTTTGTGCACGCGCGGTTTCGCGCCAAAATATTGTGACAGTGACTTTTTGTGATCAG 180

[….]

TIR_Mariner419 ------------------------------------------------------------ 0

TIR_hAT2824 ---------------------------------------------CAGTAGTTCCCAAAC 15

LOC110376033 GCCTCAGCTAACCGAAGACTGCAGAGACCAGTGCTTTATTTAGAGCAGTAGTTCCCAAAC 1500

TIR_Mariner419 ------------------------------------------------------------ 0

TIR_hAT2824 TTATTTTTCTCGTGGACCACTTTCAAAATTTTACTGGTTTCGGTGGACCCCCTGCTGCTA 75

LOC110376033 TTATTTTTCTCGTGGACCACTTTCAAAATTTTACTGGTTTCGGTGGACCCCCTGCTGCTA 1560

TIR_Mariner419 ------------------------------------------------------------ 0

TIR_hAT2824 CATTTCTACCACATTCTTAAAGTCGCCAAAAAATAAAAATTGTGTCACTTCTGAGTTCTG 135

LOC110376033 CATTTCTATCACATTCTTAAAGTCGCCAAAAAATAAAAATTGTGTCACTTCTGAGTTCTG 1620

TIR_Mariner419 ------------------------------------------------------------ 0

TIR_hAT2824 TCCCTATATTCCGATATGTAAAATAAAAACGAAAAATGGTACATTTTATATCACTTTATT 195

LOC110376033 TCCCTATATTCCGATATGTAAAATAAAAACGAAAAATGGTACATTTTATATCACTTTATT 1680

TIR_Mariner419 ------------------------------------------------------------ 0

TIR_hAT2824 TATTAAAAGAATAGAATAAGACAAGAAAAAAATATTAAGGTAATAAGTATAGGTTCAGGT 255

LOC110376033 TATTAAAAGAATAGAAGAAGACAAGAAAAAAATATTAAGGTAATAAGTATAGGTTCAGGT 1740

TIR_Mariner419 ------------------------------------------------------------ 0

TIR_hAT2824 CATAATTTTAATTAATGGGAAGGATGTATCTGATGGAGTGTCAGCAAACGATCAATGTTT 315

LOC110376033 CATAATTTTAATTAATGGGAAGGATGTATCTGATGGAGTGTCAGCAAACGATCAATGTTT 1800

TIR_Mariner419 ------------------------------------------------------------ 0

TIR_hAT2824 GGCTTTATTTTTGTGAGCAATAACCGCAAATCCCCCCGCTCTGTGATGTTTAATTTGCTC 375

LOC110376033 GGCTTTATTTTTGTGAGCAATAACCGCAAATCCCCCCGCTCTGTGATGTTTAATTGGCTC 1860

TIR_Mariner419 ------------------------------------------------------------ 0

TIR_hAT2824 CTTTTTTTTGTTAAGAGGTTTGTAACGGCACTAAAACTCCTTTCGACAAGGTATGACGAG 435

LOC110376033 CTTTTTTTTGTTAAGAGGTTTGTAACGGCACTAAAACTCCTTTCGACAAGGTATGGCGAG 1920

TIR_Mariner419 ------------------------------------------------------------ 0

TIR_hAT2824 GGAAAAGCTATTAAAAATTTCCTTGCGATTTCCCACAGTCCAGGATATTTTTCGGGTATT 495

LOC110376033 GGAAAAGCTATTAAAAATTTCCTTGCGATTTCCCACAGTCCAGGATATTTTTCGGGTATT 1980

TIR_Mariner419 ------------------------------------------------------------ 0

TIR_hAT2824 TCTGCTTGCAGCCAAAATGTTGGGTAGCCTTTTCTAAATTTCACCTTCAGCTCCTCATTA 555

LOC110376033 TCTGCTTGCAGCCAAAATGTTGGGTAGCCTTTTCTAAATTTCACCTTCAGCTCCTCATTA 2040

TIR_Mariner419 ------------------------------------------------------------ 0

TIR_hAT2824 GTGCTTAGCTCGAGCAGCTCCTCTTGTAATACAACATTGGCCACTTCCGTTTCATCAAAT 615

LOC110376033 GTGCTTAGCTCGAGCAGCTCCTCTTGTAATACAACATTGGCCACTTCCGTTTCATCAAAT 2100

TIR_Mariner419 ------------------------------------------------------------ 0

TIR_hAT2824 GGATTTATGATCCATGGTGGTATATCCATCGTCAGTATATCTTCAAACCTGGTTTGAAAT 675

LOC110376033 GGATTTATGATCCATGGTGGTATATCCATCGTCAGTATATCTTCAAACCTGGTTTGAAAT 2160

TIR_Mariner419 ------------------------------------------------------------ 0

TIR_hAT2824 TGTACGTAATATCGAATACGCAAGTTTGGACAAGTAACAGTCGCTTGCCTTATTAAAATA 735

LOC110376033 TGTACGTAATATCGAATACGCAAGTTTGGACAAGTAACAGTCGCTTGCCTTATTAAAATA 2220

TIR_Mariner419 ------------------------------------------------------------ 0

TIR_hAT2824 TTATCTGCTTAGATAGGTACTTAAAACAATGTAGGTAGGCCCTTATAAAAACTATGCTTA 795

LOC110376033 TTATCTGCTTAGATAGGTACTTAAAACAATGTAGGTAGGCCCTTATAAAAACTATGCTTA 2280

TIR_Mariner419 ------------------------------------------------------------ 0

TIR_hAT2824 CATTTTTGCTCTTTACTATCAAAAGCAGAAAAATTTTCGTGGACCCCCATTAACATCTTA 855

LOC110376033 CATTTTTGCTCTTTACTATCAAAAGCAGAAAAATTTTCGTGGACCCCCATTAACATCTTA 2340

TIR_Mariner419 ------------------------------------------------------------ 0

TIR_hAT2824 TGGACCCCCATTTTTTGTTCACGCCTACGTAGACCCCCAGCAAGTCTCCCGTGGACCCCT 915

LOC110376033 TGAACCCCCATTTTTTGTTCACGCCTACGTAGACCCCCAGCAAGTCTCCCGTGGACCCCT 2400

TIR_Mariner419 ------------------------------------------------------------ 0

TIR_hAT2824 GGGGGTCCACCTGGACCACTTTGGGAATCACTGATT------------------------ 951

LOC110376033 GGGGGTCCACCTGGACCACTTTGGGAATCACTGATTTAGAGCGACTACCTATCTGAAGTC 2460

TIR_Mariner419 ------------------------------------------------------------ 0

TIR_hAT2824 ------------------------------------------------------------ 951

LOC110376033 CTTCAAACCGTTACCGGGGCCTTTTTGCTTTTTCAGTCCCGTTGTCCCGTTGTACGCTTA 2520

[….]

TIR_Mariner419 ------------------------------------------------------------ 0

TIR_hAT2824 ------------------------------------------------------------ 951

LOC110376033 TTTAAGCATGTTCCAGACATGTTATACAGGATTCATGGCTGGGTTTCTTGCAGGCCAGTC 91980

TIR_Mariner419 -----------------CCTCGAACAAGTAATCGGTTACGCAAAGAGCTGTGTGAGGTCG 43

TIR_hAT2824 ------------------------------------------------------------ 951

LOC110376033 CAATTTTTGAATACCGACCTCGAACAAGTAATCGGTTACACAAAGAGCTGCGTGAGGTCG 92040

TIR_Mariner419 AGCATTGTCCTGCATTATACGAAATTCTTCACTTCCTATGAATCCCTGACTAGGGTAAAA 103

TIR_hAT2824 ------------------------------------------------------------ 951

LOC110376033 AGCATTGTCCTGCATTATACGAAAATATTCACTTCCTATGAATCCCTGACA-GGGTAAAA 92099

TIR_Mariner419 CATGATTTTGAAGGATCTCTTCAATATACCGCACTGTTGTCAGACGACTTTCGACAACCA 163

TIR_hAT2824 ------------------------------------------------------------ 951

LOC110376033 CATGATTTTGAAGGATTTCTTCAATATACAGCACTATTGTCAGACGATTTTCGACAACCA 92159

TIR_Mariner419 ATAATTCAGTACGGGCTTCTGAACTTATGCCTCGCCAAACCAAGATGGACCCACCATGAA 223

TIR_hAT2824 ------------------------------------------------------------ 951

LOC110376033 ATAATTCAGTACGGGATTCTGAACTTATGCCTCGCCAAACCAAAACGGACCCACCATGAA 92219

TIR_Mariner419 AACCGACTGTTTGCTTGGTAGTGGTGGGAAGAAACCATTCTCCGCGCTTTCTCCATTCAC 283

TIR_hAT2824 ------------------------------------------------------------ 951

LOC110376033 AACCGACTGTTTGCTTGGTAGTAGTGGGAAGAAGCCGTTCTCCGCGCTTTCTCCATTCAC 92279

TIR_Mariner419 ATTCACGGCCGTCTGGAGCTCTTAAGACGACTCTGCATTCATCGGTCCATAAAATTTTAC 343

TIR_hAT2824 ------------------------------------------------------------ 951

LOC110376033 ATTTACGGCCGTCTGGAGCTATTAAGACGACTCTGCATTCATCGGTCCATAAAATTTTAC 92339

TIR_Mariner419 TCCATTGGTCATGCGTCCAATTTGCATGTTCTCTTGCAAACCTAAGTCTGGCTATGCGAT 403

TIR_hAT2824 ------------------------------------------------------------ 951

LOC110376033 TCCATTGGTCATGCGTCTAATTTGCATGTTTTCTTGCAAACCTAAGTCTGGCTATGCGAT 92399

TIR_Mariner419 GGTGCGGGAGAAGTTCCGGGCCTCGAGTTGGTCTTCGAGCACGCAGATCCCGTTCCTCCA 463

TIR_hAT2824 ------------------------------------------------------------ 951

LOC110376033 AGTGCGGGAGAAGTTCCGGGCCTCGAGTTGGTCTTCGAGCACGCAGATCCCGTTCCTCCA 92459

TIR_Mariner419 TCCTTCTTCTTATTGTGCACTTACGGACGTTGACTTCTCTTGCTGTTTGCAAAGGCTGGC 523

TIR_hAT2824 ------------------------------------------------------------ 951

LOC110376033 TCCTTCTTCTTATTGTGCGCTTACGGACATTGACTTCTCTTGCTGTTTGCAAAGCCTGGC 92519

TIR_Mariner419 GTATCTCTAACGCAGTGAGAAACCGATTTTTTCATTATTGCACGTACGATAAATCGGTCG 583

TIR_hAT2824 ------------------------------------------------------------ 951

LOC110376033 GTATCTCTAACGCAGTGAGAAACCGATT-TTTCATTATTGCACGTACGATAAATCGGTCG 92578

TIR_Mariner419 TCGTGCGCCGACGAACACCTTACACCCCCACTTTCTGGTCTTCTTGTGTAAAGGCCAGTC 643

TIR_hAT2824 ------------------------------------------------------------ 951

LOC110376033 TCGTGCGCCGACGAACACCTTACACC-CCACTTTCTGGTCTTCTTGTGTAAAGGCCAGTC 92637

TIR_Mariner419 TGGTCATACATTTTCTATGCATATCTTTAACTTGTACGCGGTGCACTTAAAGTTTCAACC 703

TIR_hAT2824 ------------------------------------------------------------ 951

LOC110376033 TGGTCATACATTTTCTATGCATATCTTTAACTTGTACGCGGCGCACTTAAAGTTTCAACC 92697

TIR_Mariner419 ATCTTCCGCTGCGTACGCCCTTGACGTCTTAGCAACACTACTTGAGCAACTTGAGCTGCA 763

TIR_hAT2824 ------------------------------------------------------------ 951

LOC110376033 ATCTTCCACTGCTTCCGCCCTTGACGTCTTAGCAACACTACTTGAGCAACTTGAGCTGCA 92757

TIR_Mariner419 GTAAGGGTAATTTTCAGATCAAATTGTGAAAAAAAAGAGAAACAAAAAACGATCATAATC 823

TIR_hAT2824 ------------------------------------------------------------ 951

LOC110376033 GTAAGGGTAATTTTCAGATCAAATTGTGAAAAAAAGAGAAAC-AAATAACGATCATAAAC 92816

TIR_Mariner419 ATTATTTTTTTTTTTGGAACGTGCTTAGTACTTCGGCAATTTCTATCTGAATTTAAACTT 883

TIR_hAT2824 ------------------------------------------------------------ 951

LOC110376033 A--TAAATTTTTTTTGGAACGTGCTTAGTACTTCAGCAATTTCTATCTGAATTTAAACTT 92874

TIR_Mariner419 AACTTTCTGCTTTGTGTTCCAACAACGGAATCTGTTTCTAGTGTTATAAAAGTTAAACAG 943

TIR_hAT2824 ------------------------------------------------------------ 951

LOC110376033 AACTTTCTGCTTTGTGTTCCAATAAAGGAAGCTGTTTCTAGTGTTACAAAAGTTAAACAG 92934

TIR_Mariner419 ACACACATTTTTCTGTATTTTAATTAACAATTACGAAAGGACTGACAATATGTCATATGA 1003

TIR_hAT2824 ------------------------------------------------------------ 951

LOC110376033 ACAGACATTTTTCTGTATTTTAATTAACAATTACGAAAGGACTGACAATATGTCATATGA 92994

TIR_Mariner419 AATTAGAATTTACAACAGCTATCTGGGCTGATATCTACTTGTGTTTGTTTGGGCCAAATT 1063

TIR_hAT2824 ------------------------------------------------------------ 951

LOC110376033 AATTAGAATTTACAACAGCTATCTGGGCTGATATCTACTTGTATTTGTTTGGGCCAAATT 93054

TIR_Mariner419 CCGTGCCGCTGAGTGTAGTTTC-------------------------------------- 1085

TIR_hAT2824 ------------------------------------------------------------ 951

LOC110376033 CCGTGCCGCTGAGTGTAATTAATTATACAAATAGTCCTTTTTCTAATTTTGAATTCTGTA 93114

TIR_Mariner419 ------------------------------------------------------------ 1085

TIR_hAT2824 ------------------------------------------------------------ 951

LOC110376033 AACTGCGTTATATTGATTTAACAATAGAAACTATTTGAAAAACCACTTTCGGCTTCAAGT 93174

**Figure S7. Alignment of TIR_mariner419 and TIR_hAT824 elements with ABCG member 20 gene**
